# Evaluation of the Immunochromatographic NG-Test Carba 5, RESIST-5 O.O.K.N.V., and IMP *K*-SeT for Rapid Detection of KPC-, NDM-, IMP-, VIM-type, and OXA-48-like Carbapenemase Among *Enterobacterales*

**DOI:** 10.3389/fmicb.2020.609856

**Published:** 2021-01-15

**Authors:** Renru Han, Yan Guo, Mingjia Peng, Qingyu Shi, Shi Wu, Yang Yang, Yonggui Zheng, Dandan Yin, Fupin Hu

**Affiliations:** ^1^Institute of Antibiotics, Huashan Hospital, Fudan University, Shanghai, China; ^2^Key Laboratory of Clinical Pharmacology of Antibiotics, Ministry of Health, Shanghai, China

**Keywords:** immunochromatographic assay, carbapenem-resistant *Enterobacterales*, carbapenemase, NG-test Carba 5, RESIST-5 O.O.K.N.V, IMP K-SeT

## Abstract

**Background:**

*Enterobacterales* are the most common pathogens for nosocomial infections. The emergence and spread of KPC, NDM, and OXA-48-like carbapenemase-producing *Enterobacterales* with their extensively drug-resistant characteristics have posed great threats to public health. This study aimed to evaluate the performance of NG-test Carba 5, RESIST-5 O.O.K.N.V., and IMP *K*-SeT for rapid detection of five carbapenemases (KPC, NDM, VIM, IMP, and OXA-48-like) among *Enterobacterales*.

**Methods:**

A total of 186 carbapenem-resistant *Enterobacterales* clinical isolates and 29 reference strains were used in this study. Carbapenemase genes were confirmed by PCR and DNA sequencing. The sensitivities and specificities of these assays were calculated utilizing the VassarStats software.

**Results:**

For clinical isolates, the NG-test Carba 5 detected KPC, NDM, OXA-48-like, IMP, and VIM in less than 15 min with the sensitivity and specificity of 100% and 100%, respectively. The RESIST-5 O.O.K.N.V. detected KPC, NDM, OXA-48-like, and VIM with the sensitivity and specificity of 99.4 and 100%. The IMP *K*-SeT detected all of the IMP producers (6/6). For reference strains, the sensitivity and specificity of NG-test Carba 5, RESIST-5 O.O.K.N.V., and IMP *K*-SeT were all 100 and 100%, respectively.

**Conclusion:**

As efficient, rapid, and convenient diagnostic methods, NG-test Carba 5, RESIST-5 O.O.K.N.V., and IMP *K*-SeT could help to simplify the complex routine workflow for detecting carbapenemases. Rapid and accurate identification of carbapenemase is of significance for both epidemiological and infection control purposes.

## Introduction

The emergence and dissemination of carbapenemase-producing *Enterobacterales* (CPE) pose a global health threat (Nordmann et al., 2012a). As the ability of carbapenemases to hydrolyze all β-lactam antibiotics leads to few antibiotics retaining activity against CPE, infections caused by CPE are usually burdened by high mortality and poor prognoses (Falagas et al., 2014; Feil, 2016). Tigecycline, colistin, and ceftazidime-avibactam are the only available antimicrobial agents for the treatment of infections caused by CPE in China. However, unlike tigecycline and colistin, the activity of ceftazidime-avibactam is varied to different CPE. The *in vitro* studies show that ceftazidime-avibactam has excellent *in vitro* activity against ESBL-, AmpC-, KPC-, and OXA-48-producing *Enterobacterales*, but has no activity against metallo-beta-lactamases *Enterobacterales* (Shirley, 2018; Yin et al., 2019). As reported, for the treatment of carbapenem-resistant *Klebsiella pneumoniae* bloodstream infections, initial adequate antibiotic therapy resulted in the only independent factor able to protect against death (Micozzi et al., 2017). Therefore, rapid and accurate identification of carbapenemases is critical for both epidemiological and infection control purposes.

Recently, rapid diagnostic tests, NG-test Carba 5 immunochromatographic assay (NG Biotech, Guipry, France), and RESIST-5 O.O.K.N.V. (CORIS, BioConcept, Gembloux, Belgium) have been developed to detect the five main carbapenemases, namely, KPC, NDM, OXA-48-like, IMP, and VIM. To date, several studies have evaluated the performance of NG-test Carba 5 and demonstrated that it performed well on bacterial colonies and positive blood cultures with overall sensitivity and specificity that ranged from 97.3 to 100% and 95.3 to 100%, respectively (Boutal et al., 2018; Hopkins et al., 2018; Bodendoerfer et al., 2019; Giordano et al., 2019). The RESIST-5 O.O.K.N.V. detected KPC-type and OXA-48-like carbapenemases from blood cultures with a sensitivity of 100% for both, but 50.0 and 52.2% for NDM- and VIM-type carbapenemases, respectively (Bianco et al., 2020). Comparative studies evaluating the performance of different lateral flow chromatographic assays to detect carbapenemase from bacterial colonies are lacking in China. In this study, we investigated the performance of NG-test Carba 5, RESIST-5 O.O.K.N.V., and IMP *K*-SeT assay to detect carbapenemases among CPE.

## Materials and Methods

### Strains

From January 2016 to December 2018, 186 non-duplicate clinical isolates were collected from 32 hospitals of 22 provinces or cities across China, and 29 reference strains were purchased from the American Type Culture Collection^1^. These clinical isolates were resistant to at least one of the carbapenem antibiotics (ertapenem, meropenem, or imipenem), including 124*K. pneumoniae*, 26 *Escherichia coli*, 23 *Enterobacter cloacae*, 5 *Klebsiella oxytoca*, 2 *Citrobacter freundi*, 2 *Enterobacter aerogenes*, 2 *Serratia marcescens*, 1 *Morganella morganii*, and 1 *Providencia rettgeri*. Twenty-nine reference strains (including 17 *K. pneumoniae*, 8 *E. coli*, 1 *E. cloacae*, 1 *K. oxytoca*, 1 *P. rettgeri*, and 1 *Enterobacter hormaechei*) with or without carbapenemase were involved in this study (Table 1). All tested isolates were identified to the species level using matrix-assisted laser desorption/ionization-time of flight mass spectrometry (bioMérieux, France).

**TABLE 1:** Reference strains used in this study.

**Reference Strains**	**Species**	**β-lactamase genes**	**NG-test Carba 5**	**RESIST-5O.O.K.N.V. /IMP *K*-SeT**
ATCC BAA 2146	*K. pneumoniae*	NDM-1	NDM	NDM
ATCC BAA 2452	*E. coli*	NDM-1	NDM	NDM
ATCC BAA 2469	*E. coli*	NDM-1	NDM	NDM
ATCC BAA 2470	*K. pneumoniae*	NDM-1	NDM	NDM
ATCC BAA 2471	*E. coli*	NDM-1	NDM	NDM
ATCC BAA 2473	*K. pneumoniae*	NDM-1	NDM	NDM
ATCC BAA 1705	*K. pneumoniae*	KPC-2	KPC	KPC
ATCC BAA 1899	*K. pneumoniae*	KPC-2	KPC	KPC
ATCC BAA 1900	*K. pneumoniae*	KPC-2	KPC	KPC
ATCC BAA 1902	*K. pneumoniae*	KPC-2	KPC	KPC
ATCC BAA 1903	*K. pneumoniae*	KPC-2	KPC	KPC
ATCC BAA 1904	*K. pneumoniae*	KPC-2	KPC	KPC
ATCC BAA 1905	*K. pneumoniae*	KPC-2	KPC	KPC
ATCC BAA 2341	*E. cloacae*	KPC-2	KPC	KPC
ATCC BAA 2342	*K. pneumoniae*	KPC-2	KPC	KPC
ATCC BAA 2343	*K. pneumoniae*	KPC-2	KPC	KPC
ATCC BAA 2344	*K. pneumoniae*	KPC-2	KPC	KPC
ATCC BAA 2082	*E. hormaechei*	KPC-2	KPC	KPC
ATCC BAA 2340	*E. coli*	KPC-2	KPC	KPC
ATCC BAA 2078	*K. pneumoniae*	KPC-2	KPC	KPC
ATCC BAA 2523	*E. coli*	OXA-48	OXA	OXA
ATCC BAA 2524	*K. pneumoniae*	OXA-48	OXA	OXA
ATCC BAA 2525	*P. rettgeri*	OXA-181	OXA	OXA
ATCC 51983	*K. oxytoca*	ESBL	NEG	NEG
ATCC BAA 204	*E. coli*	ESBL	NEG	NEG
ATCC BAA 198	*E. coli*	ESBL	NEG	NEG
NCTC 13440	*K. pneumoniae*	VIM-1	VIM	VIM
NCTC 13476	*E. coli*	IMP-1/70	IMP	IMP
NCTC 13439	*K. pneumoniae*	VIM-1	VIM	VIM

### Detection of Carbapenemase genes

For 186 carbapenem-resistant *Enterobacterales* (CRE) clinical strains, carbapenemase genes (*bla*_KPC_, *bla*_NDM_, *bla*_OXA–48–like_, *bla*_IMP_, and *bla*_VIM_) were detected by polymerase chain reaction (PCR) with specific primers and conditions as described previously (Poirel et al., 2011). The positive PCR amplicons were sequenced and compared with the reported sequences from GenBank by Blast^2^. As a result, 172 were positive for carbapenemase genes, including *bla*_KPC–2_ (*n* = 70), *bla*_NDM–1_ (*n* = 35), *bla*_NDM–5_ (*n* = 23), *bla*_NDM–7_ (*n* = 1), *bla*_NDM–9_ (*n* = 1), *bla*_OXA–232_ (*n* = 20), *bla*_OXA–181_ (*n* = 2), *bla*_OXA–48_ (*n* = 2), *bla*_IMP–4_ (*n* = 5), *bla*_IMP–6_ (*n* = 1), *bla*_VIM_ (*n* = 1), *bla*_KPC–2_ plus *bla*_NDM–1_ (7/7), *bla*_KPC–2_ plus *bla*_NDM–5_ (1/1), *bla*_NDM–5_ plus *bla*_OXA–48_ (1/1), *bla*_NDM–1_ plus *bla*_IMP–4_ (1/1), and *bla*_NDM–1_ plus *bla*_IMP–6_ (1/1). Fourteen CRE clinical strains were carbapenemase-negative.

### Immunochromatographic Assays

The NG-Test Carba 5 assay consists of an independent cassette (targets KPC-, NDM-, VIM-, and IMP-type and OXA-48-like five main carbapenemases). RESIST-5 O.O.K.N.V. consists of two independent K-SeTs (one for the detection of OXA-163, OXA-48-like, and KPC; another for the detection of VIM and NDM). Both cassettes are provided in a single package and are to be used in parallel on the same bacterial lysis preparation. IMP K-SeT consists of a K-SeT for the detection of IMP metallo-β-lactamase, which was performed as a complementary test of RESIST-5 O.O.K.N.V.

These tests were performed according to the manufacturer’s instructions in parallel. Firstly, one single isolated colony of overnight growth was harvested from the plate to an Eppendorf tube or tube with extraction buffer and suspended thoroughly to perform the lysis step. Subsequently, approximately 100 μl of the mixture was loaded on the sample region of the cassette and allowed to migrate for 15 min. Finally, the results were read until the control line turned red in the control region and then recorded whether the lines turned red in the test region of the cassette 15 min later.

### Statistical Analysis

The sensitivity and specificity of the assay and upper and lower limits of the 95% confidence intervals (CIs) were calculated utilizing the VassarStats software^3^.

## Results

The NG-test Carba 5 and RESIST-5 O.O.K.N.V. showed the detection of 100% of KPC-2 (70/70), NDM-5 (23/23), NDM-7 (1/1), VIM (1/1), OXA-232 (20/20), OXA-181 (2/2), and OXA-48 (2/2) with no false positive. This study also demonstrated that 100% of IMP-4 (5/5) and IMP-6 (1/1) were correctly detected by NG-test Carba 5 and IMP *K*-SeT. NG-test Carba 5 showed the detection of 100% of NDM-1 (35/35), whereas RESIST-5 O.O.K.N.V. showed the detection of 97.1% of NDM-1 (34/35) except one *P. rettgeri*. Moreover, NG-test Carba 5 and RESIST-5 O.O.K.N.V. were able to detect double carbapenemases-producers simultaneously, and the results showed 100% of KPC-2 plus NDM-1 (7/7)-, KPC-2 plus NDM-5 (1/1)-, and NDM-5 plus OXA-48 (1/1)-producing isolates with two red positive lines in the test region. All of the non-carbapenemase (0/14) producers were negative in the tests (Table 2). Overall, the sensitivity and specificity of NG-test Carba 5 to detect five main carbapenemases were both 100%. The sensitivity and specificity of RESIST-5 O.O.K.N.V. were 99.4 and 100%, respectively (Table 2).

**TABLE 2:** Rapid identification of carbapenemases by NG-test Carba 5, RESIST-5 O.O.K.N.V., and IMP *K*-SeT.

**Strains**	**β -lactamase**	**No. of positive tests/total no. of strains**			**Sensitivity [% (95% CI)]**	**Specificity [% (95% CI)]**
		**NG-test Carba 5**	**RESIST-5 O.O.K.N.V.**	**IMP *K*-SeT**		
**Clinical strains**						
	KPC-2	70/70	70/70	0/70	100 (93.5–100)	100 (96.0–100)
	NDM	60/60			100 (92.5–100)	100 (96.3–100)
			59/60	0/60	98.3 (89.7–100)	100 (96.3–100)
	NDM-1	35/35	34/35			
	NDM-5	23/23	23/23			
	NDM-7	1/1	1/1			
	NDM-9	1/1	1/1			
	IMP	6/6	0/6	6/6	100 (51.7–100)	100 (97.4–100)
	IMP-4	5/5	0/5	5/5		
	IMP-6	1/1	0/1	1/1		
	OXA-48-like	24/24	24/24	0/24	100 (82.8–100)	100 (97.1–100)
	OXA-232	20/20	20/20			
	OXA-48	2/2	2/2			
	OXA-181	2/2	2/2			
	VIM-1	1/1	1/1	0/1	100 (5.5–100)	100 (97.5–100)
	Multiple carbapenemases	11/11	11/11	2/11	100 (67.9–100)	100 (97.3–100)
	KPC-2 + NDM-1	7/7	7/7			
	KPC-2 NDM-5	1/1	1/1			
	NDM-5 OXA-48	1/1	1/1			
	NDM-1 IMP-4	1/1	1/1			
	NDM-1 IMP-6	1/1	1/1			
	Carbapenemase-negative	0/14	0/14	0/14		
	Total	172/186			100 (97.3–100)	100 (73.2–100)
			165/186	6/186	99.4 (96.2–100)	100 (80.0–100)
**Reference strains**						
	KPC-2	14/14	14/14	0/14		
	NDM-1	6/6	6/6	0/6		
	IMP-1/70	1/1	0/1	1/1		
	VIM-1	2/2	2/2	0/2		
	OXA-48-like	3/3	3/3	0/3		
	OXA-48	2/2	2/2			
	OXA-181	1/1	1/1			
	ESBL	0/3	0/3	0/3		
	Total	26/29			100 (84.0–100)	100 (31.0–100)
			25/29	1/1	100 (83.4–100)	100 (39.6–100)

For the 29 reference strains, NG-test Carba 5, RESIST-5 O.O.K.N.V., and IMP *K*-SeT detected all KPC-2 (14/14), NDM-1 (6/6), OXA-48 (2/2), OXA-181 (1/1), VIM-1 (2/2), and IMP-1 producers (1/1) (Table 1). Three ESBL producers were negative without cross-reaction. The overall sensitivity and specificity of NG-test Carba 5, RESIST-5 O.O.K.N.V., and IMP *K*-SeT detecting carbapenemases were all 100 and 100%, respectively (Table 2).

## Discussion

Of note, the rapid detection and identification of carbapenemase can help to prevent the spread and infection control of carbapenemase-producing isolates in health facilities (Boutal et al., 2018; Takissian et al., 2019). A nationwide survey indicated that *bla*_KPC–2_ (57% and 627/1,105) and *bla*_NDM_ (31% and 343/1,105) were the most common carbapenemase genes among carbapenem-resistant *Enterobacterales* clinical isolates in China (Zhang et al., 2017; Han et al., 2020). Rapid identification of the carbapenemase type can help to guide therapy for the treatment of infection caused by carbapenemase-producing isolates. As reported, most KPC-2 or OXA-48-like carbapenemase-producing isolates are susceptible to ceftazidime-avibactam (Falcone and Paterson, 2016; Bassetti et al., 2018; Stewart et al., 2018; Zou et al., 2019). On the contrary, ceftazidime-avibactam has no activities against metallo-β-lactamase (NDM-, VIM-, or IMP-type) producers; thus other therapeutic options have to be considered, such as tigecycline, colistin, or other available antimicrobial agents (Davido et al., 2017; Zusman et al., 2017; Jayol et al., 2018; Emeraud et al., 2019; Yin et al., 2019; Zou et al., 2019).

Currently, several phenotypic methods have been developed for the detection and identification of carbapenemases. Modified carbapenem inactivation methods (mCIMs) had sensitivities ranging from 93 to 100% and specificities from 97 to 100% for the detection of CPE, and excellent reproducibility was shown across laboratories (Pierce et al., 2017; Tsai et al., 2020). The mCIM and EDTA-CIM detected metallo-carbapenemases with a sensitivity of 89.3% and a specificity of 98.7% (Tsai et al., 2020). The mCIM was easy to perform and interpret for *Enterobacterales*, but time-consuming (overnight incubation). Besides, invalid or uncertain results may occur with some isolates, and certain carbapenemase types are not detected consistently.

Carba NP test detected most carbapenemases with high sensitivities from 73 to 100% among *Enterobacterales*, but with low sensitivity in detecting OXA-48-like carbapenemase producers (Nordmann et al., 2012b; Tijet et al., 2013; Vasoo et al., 2013; Yusuf et al., 2014; Papagiannitsis et al., 2015; Tamma et al., 2017). The modified Carba NP test had a sensitivity of 99% (Tamma et al., 2017). One limitation of the manual versions of the Carba NP test and its variants was frequent reagent preparation due to the short shelf life of the imipenem-containing solution. Other commercially available assays, including the Rapidec Carba NP test, the Neo-Rapid Carb screen, and the Rapid Carb Blue screen have sensitivities ranging from 89 to 98% and specificities approaching 100% (Tamma et al., 2017). Similar to the Carba NP test, the limitations of these commercial assays are as follows: false-negative results occurred with OXA-48-like carbapenemase and the interpretation of results can be subjective due to slight color changes (Tamma and Simner, 2018). The sensitivites of the modified Hodge test (MHT) have been reported to be between 93 and 98% in KPC producers, but low sensitivites for metallo-beta-lactamases (Girlich et al., 2012; Mathers et al., 2013; Vasoo et al., 2013; Tsai et al., 2020). Reported sensitivities and specificities of MALDI-TOF MS ranged from 77 to 100% and 94 to 100%, respectively, with a turnaround time of within 4 h (Knox et al., 2014; Papagiannitsis et al., 2015; Oho et al., 2020). Carbapenemase inhibition tests with boronic acid derivatives (BA) and dipicolinic acid (DPA)/EDTA were tested in *Enterobacterales*. The sensitivity for identification of class A, B, and OXA-48 carbapenemases was 95, 90, and 100%, with 96 to 100% specificity (van Dijk et al., 2014).

Compared with the phenotypic method, the multiplex immunochromatographic assay for the detection of KPC-, NDM-, VIM-, IMP-, and OXA-48-like carbapenemases was easy to perform and only relatively little hands-on time (no more than 5 min) was required. Several studies assessing the immunochromatographic for the detection of common carbapenemases showed high sensitivity and specificity results in bacteria (Boutal et al., 2018; Hopkins et al., 2018; Bodendoerfer et al., 2019). The NG-test Carba 5 has evaluated the detection of CPE from spiked blood cultures with a sensitivity and specificity of 97.7 to 98.3% and 96.1 to 100%, respectively (Giordano et al., 2019; Takissian et al., 2019). The RESIST-5 O.O.K.N.V. detected KPC-type and OXA-48-like carbapenemases from blood cultures with a high sensitivity of 100%, but with low sensitivities of 50.0 and 52.2% in detecting NDM- and VIM-type carbapenemases, respectively, and the results were quite different from our research of NDM metallo-beta-lactamases (97.1% sensitivity) (Bianco et al., 2020).

The results of our study indicated that the NG-test Carba 5 showed the detection of 100% of KPC-2, NDM, VIM, and OXA-48-like with no false positive. The RESIST-5 O.O.K.N.V. showed 100% detection of KPC-2, OXA-48-like, IMP, and VIM, whereas it showed 97.1% detection of NDM-1 (34/35) with one false-negative result. We speculated that this might be associated with the mean intensity of NDM bands; NG-Test Carba 5 was 2.2 times higher than RESIST-4 O.K.N.V. (Bogaerts et al., 2020). Additionally, NG-test Carba 5, RESIST-5 O.O.K.N.V., and IMP *K*-*SeT* were also able to detect two carbapenemases simultaneously with a sensitivity of 100%. Of concern, NG-test Carba 5 was not able to distinguish OXA-48-like variants (OXA- 48-, OXA- 181-, OXA- 163-, OXA- 232-, and OXA-405-type carbapenemase), and the OXA-163- and the OXA-405-producing strains might be considered as false-negative results for OXA-48-like carbapenemase producers (Boutal et al., 2018; Takissian et al., 2019). In this case, the RESIST-5 O.O.K.N.V. assay could fill this gap by specifically detecting OXA-163 (OXA-48-like variants) with a sensitivity of 100%, which was an updated version of the RESIST-3 O.O.K. *K*-SeT (Coris BioConcept) and the OXA-163/48 *K*-SeT (Coris BioConcept) (Meunier et al., 2016; Pasteran et al., 2016). Due to a lack of OXA-163 producers, we could not evaluate the ability of RESIST-5 O.O.K.N.V. in detecting OXA-163. The limited detection ranges of these assays cover only common carbapenemases, which lead to the neglect of rare carbapenemases like GES, IMI, and GIM.

Our study had three limitations. The first limitation is including only six *bla*_IMP_-positive strains and one *bla*_VIM_-positive strain. Second, there is a lack of other KPC- and OXA-48-like variant carbapenemases such KPC-3 and OXA-163 in this study; these variants are rare in China. Third, limited non-CPE isolates were used to assess the specificity of the assays, and we need to sufficiently investigate this isolates to evaluate the performance of these assays in this study. Recently, we collected one *K. pneumoniae* harboring a new variant, rare carbapenemase *bla*_KPC–33_ (Shi et al., 2020), and we found that immunochromatographic assays cannot detect this new variant. Although other *bla*_KPC_-positive strains including *bla*_KPC–3_-, *bla*_KPC–33_-, or *bla*_VIM_-type metallo-β-lactamase positive strains are rare in China, we still need to collect these strains to make a more comprehensive assessment on the performance of immunochromatographic testing for detection of carbapenemases.

## Conclusion

In conclusion, NG-test Carba 5, RESIST-5 O.O.K.N.V., and IMP *K*-SeT assays could be efficient, rapid, and convenient diagnostic tools for detecting the most common carbapenemases in China. These might help to control the spread of carbapenemase-producing isolates in health facilities.

## Data Availability Statement

The original contributions presented in the study are included in the article/supplementary material, further inquiries can be directed to the corresponding author/s.

## Ethics Statement

The study protocol was approved by the Institutional Review Board of Huashan Hospital, Fudan University (Number: 2018-408).

## Author Contributions

FH designed the study. RH, YG, MP, QS, and SW performed the experimental work. RH, YG, YY, and DY collected the data. FH and RH analyzed the data. All authors read and approved the final manuscript.

## Conflict of Interest

The authors declare that the research was conducted in the absence of any commercial or financial relationships that could be construed as a potential conflict of interest.

## Footnotes

1 https://www.atcc.org

2 www.ncbi.nlm.nih.gov/blast/

3 http://www.vassarstats.net/
